# Supplementary material for: Incidence rate, risk factors, and management of Bell’s palsy in the Qurayyat region of Saudi Arabia
Source: PeerJ. 2022 Oct 6;10:e14076. doi: 10.7717/peerj.14076 (PMC9548320; doi:10.7717/peerj.14076)
Supplement: Supplemental Information 1 [file peerj-10-14076-s001.pdf]

١. **الجنس**
- ذكر
- انثى

٢. **العمر**
- من ٢١-٣٠
- من ٣١-٤٠
- من٤١-٥٠
- من ٥١-٦٠
- من ٦١ و أكثر

٣. **كيف بدأت إصابتك بشلل الوجه؟**
- مفاجئة
- تدريجية

٤. **هل تكررت أصابتك؟**
- لا
- نعم

٥. **كم مرة تكررت إصابتك؟**
- 1
- 2
- 3
- 4 و اكثر

٦. **هل أخذت تطعيمات قبل الإصابة بشلل الوجه ؟**
- لا
- نعم

٧. **هل تعرضت لتيار هواء بارد قبل الإصابة بشلل الوجه؟**
- لا
- نعم

٨. **هل أصبت بعدوى فيروسية قبل الإصابة بشلل الوجه ؟**
- لا
- نعم

٩. **هل أصبت بنزلات برد قبل الإصابة بشلل الوجه؟**
- لا
- نعم

١٠. **هل أصبت بالإنفلونزا قبل الإصابة بشلل الوجه؟**

١١. **هل لديك ارتفاع في ضغط الدم ؟**
- لا
- نعم

١٢. **هل لديك مرض السكري؟**
- لا
- نعم

١٣. **هل لديك التهابات في الاذن ؟**
- لا
- نعم

١٤. **هل تعرضت لإصابة في الرأس ؟**
- لا
- نعم

١٥. **هل أجريت عملية في الرأس أو الرقبة أو الوجه ؟**
- لا
- نعم

١٦. **ما هو الجانب المصاب بشلل الوجه ؟**
- الايسر
- الايمن
- كلاهما

١٧. **هل هناك صلة قرابة بين الأب والأم ؟**
- لا
- نعم

١٨. **هل أصيب أحد من عائلتك بشلل الوجه ؟**
- لا
- نعم

١٩. **هل لديك أمراض وراثية ؟**
- لا
- نعم

٢٠. **هل أصبت بجلطة قبل الإصابة بشلل الوجه ؟**
- لا
- نعم

٢١. **هل لديك أمراض عصبية ؟**
- لا
- نعم

٢٢. **هل لديك أمراض او مشاكل في القلب ؟**

- لا
- نعم

٢٣. **هل لديك أمراض او مشاكل في الجهاز التنفسي ؟**
- لا
- نعم

٢٤. **هل أنت مدخن ؟**
- لا
- نعم

٢٥. **هل لديك عيوب او تشوهات خلقية في الرأس ؟**
- لا
- نعم

٢٦. **هل لديك مشاكل في العين ؟**
- لا
- نعم

٢٧. **هل لديك مشاكل في التوازن ؟**
- لا
- نعم

٢٨. **ماهي طريقة العلاج المستخدمة عندما اصبت بشلل الوجه؟**
